# Ambulatory short-term mechanical circulatory support: facilitates recovery and prepares patients for definitive therapy

**DOI:** 10.1007/s12055-023-01512-9

**Published:** 2023-05-16

**Authors:** Jaishankar Raman, Pankaj Saxena

**Affiliations:** 1 Department of Cardiothoracic Surgery, Austin Health & St Vincent’s Hospitals, University of Melbourne, Melbourne, Australia; 2 Department of Cardiothoracic Surgery, Townsville University Hospital, Townsville, Australia

**Keywords:** Mechanical circulatory support, Subclavian balloon, Ambulatory support, ECMO, Impella

## Abstract

Short-term mechanical circulatory support (ST-MCS) devices have been traditionally deployed in patients with cardiogenic shock, advanced heart failure, cardiovascular collapse, and cardiorespiratory failure. Limitations of the mechanical support devices are typically related to mobility of the patient since the access is frequently through femoral vasculature. This limits the time the patient can be supported by mechanical circulatory support (MCS). We describe deployment of ST-MCS using alternate access such as the subclavian/axillary artery that facilitates ambulation of the patient. These include the deployment of intra-aortic balloon pump (IABP) through the subclavian artery, Impella pump through the axillary/subclavian artery, and extracorporeal membrane oxygenation (ECMO) using the subclavian artery and jugular vein.


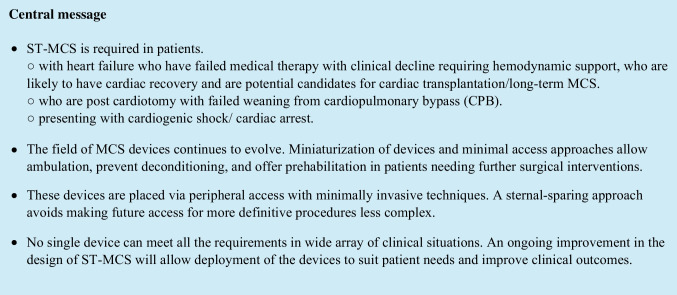


## Introduction

The field of mechanical circulatory support (MCS) has been evolving steadily as the burden of patients with cardiovascular diseases causing heart failure (HF) has continued to rise exponentially. MCS devices are broadly of two types: short-term and long-term. The short-term devices are used in the setting of cardiogenic shock following acute myocardial infarction (MI), acute massive pulmonary embolism, post cardiotomy patients with failure to wean from cardiopulmonary bypass (CPB), cardiac arrest, myocarditis, cardiomyopathy with acute decompensation, and cardiorespiratory failure [1]. Long-term devices or durable ventricular assist devices (VAD) are usually implanted in patients who are candidates for cardiac transplant and as destination therapy [2]. The latter are predominantly placed to support the left heart and require coumadin/warfarin. Either of the devices could potentially be used as bridge to recovery, as bridge to decision, and as bridge to transplant. Decision-making for type of MCS implantation could be a complex process considering patients’ primary conditions causing HF and presence of comorbidities. The decision-making for candidacy of various devices usually involves a multi-disciplinary approach. Determining MCS candidacy is often difficult and requires the integration of clinical, biomarker, imaging, exercise, and hemodynamic data. The conditions that exclude the patients from their candidacy for MCS implant include advanced malignancy, severe systemic disease with poor prognosis, neurologic injury with poor prospects of recovery, poorly controlled sepsis, frailty, and patients who have major psycho-social issues.

The number of patients who are becoming eligible for MCS is rising due to many ongoing technical advances. One of the important factors includes miniaturization of the devices which can be implanted, allowing the patients to stay ambulatory. Ambulatory devices facilitate patients’ recovery by facilitating their ability to undergo physical conditioning. This is crucial for maintaining muscle mass and allows them to recover well or undergo a major procedure like a heart transplant.

The evolution from older pulsatile devices to continuous-flow left ventricular assist devices (LVAD) and the incorporation of smaller pumps, with no valves, fewer moving parts, and improved hemocompatibility have translated into improved clinical outcomes, greater durability, fewer adverse events, and reduced overall cost of care. This review aims to provide a summary of the current use of short-term and durable MCS devices in the treatment of advanced-stage HF, highlighting several aspects of LVAD support and the challenges that remain. The intra-aortic balloon pump (IABP) has been the most commonly used temporary mechanical support device over the past 50 years [3]. The use of the subclavian artery and then the axillary artery arose from experience in aortic surgery with reduced stroke rates [4]. This was adapted to an alternate access for insertion of the balloon pump which evolved to ambulatory use at the University of Chicago in 2008 by the authors [5].

## Subclavian/axillary artery access

**Fig. 1:**
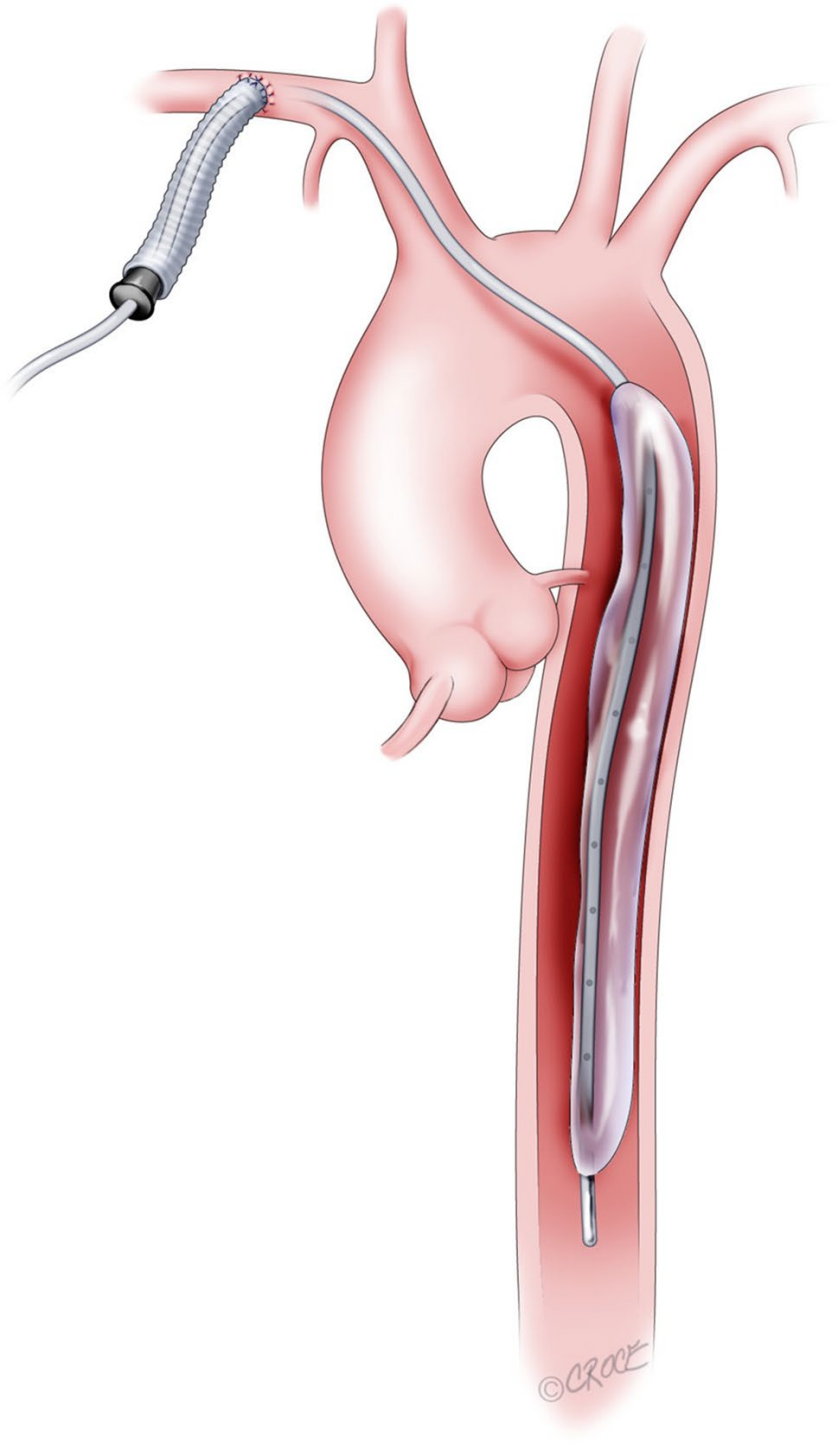
Subclavian balloon pump schematic

The subclavian/axillary artery is easily approached in the infra-clavicular region by splitting the pectoralis major muscle fibers and dividing the clavipectoral fascia. The subclavian vein is dissected out and moved downwards, to expose the subclavian artery just as it becomes the axillary artery. This first gained prominence in providing arterial inflow and antegrade cerebral perfusion in aortic surgery. This was adapted to use for implantation of IABPs through the right subclavian artery [5]. This allowed the first few patients to be bridged to transplant [6]. The longest implant in the early experience had a conventional balloon pump implanted by right subclavian access, in situ for 65 days prior to transplantation. Thereafter, a small series was reported from the University of Chicago, using this approach in high-risk patients undergoing complex cardiac surgery with encouraging results [7]. Figure 1 is a schematic depiction of the balloon pump inserted through the right subclavian artery.

**Fig. 2:**
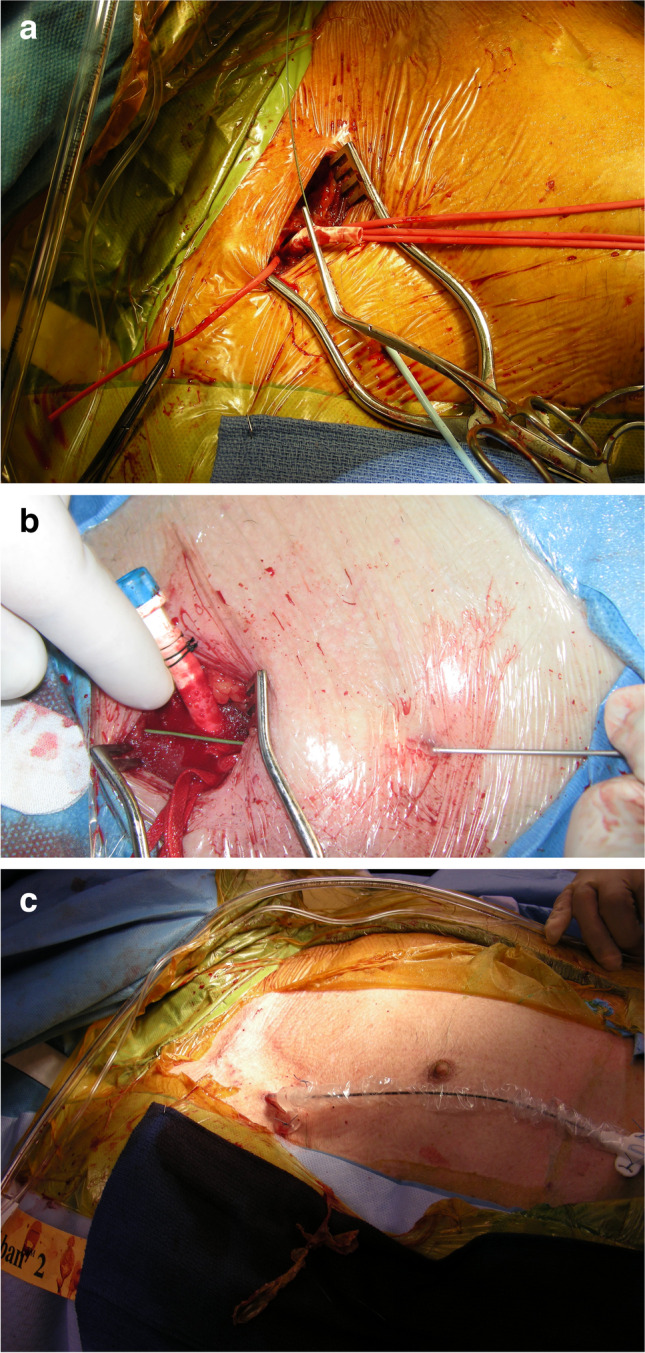
Steps of balloon inserted through the right subclavian artery. **a** Graft sutured to the subclavian artery. **b** Wire introduced with graft sealed with a one-way valve. **c** Balloon in position

Components of the operative procedure are shown in Fig. 2. This shows the Gore-Tex graft being sutured (Fig. 2a), followed by the wire being introduced (Fig. 2b). The extravascular component of the balloon pump catheter secured on the chest wall is shown in Fig. 2c.

IABP support has been the most commonly used type of temporary MCS. The device is traditionally used in stabilizing the patients with cardiogenic shock usually in the setting of acute myocardial infarction (AMI), patients with unstable angina, management of mechanical complications of MI, and post cardiotomy patients needing hemodynamic support to wean off CPB. The IABP catheter is usually inserted via a transfemoral approach percutaneously. The device works on the principle of diastolic augmentation, improving coronary perfusion and reduction in systemic vascular resistance due to sudden balloon deflation during systole, causing offloading of the left ventricle (LV). The IABP has been used in the management of HF patients as bridge to recovery, bridge to longer-term LVADs, and as bridge to transplant. Nishida et al. used the trans-axillary approach for IABP placement in 133 patients as bridge to transplant; 70.7% had a graft sutured to the axillary/subclavian artery while the rest of the patients had implants through a percutaneous approach [8]. The group demonstrated that 122 (91.7%) patients proceeded to transplant. The mean duration of the support was 21 days, suggesting that this support can reliably be used as a bridge in deteriorating patients while being on the transplant waiting list.

However, the main issue with IABP is that it still requires the use of a large external console that will keep the patient hospitalized. The incidence of migration, driveline kinking, and IABP malfunction has been higher when it has been placed via trans-axillary approach [8]. Stroke can be another uncommon problem with a subclavian balloon, most likely related to the presence of the aortic or arch vessel atheroma [9].

### Operative technique

The open technique involves exposure of the junction of the right or left axillary/subclavian artery junction via a small infraclavicular incision. A 4–7-mm polytetrafluoroethylene (PTFE) graft is sutured to the axillary/subclavian artery and the one-way valve part of the 8-Fr sheath is tied to the graft. The guidewire is introduced through the sheath. Fluoroscopic guidance is used for ensuring the placement of guidewire in the descending thoracic aorta [5, 6]. Several options are available to help direct the guidewire into the descending thoracic aorta using a range of percutaneous catheters. However, migration of the device could be a potential issue in these patients due to a significant component of the balloon catheter sitting outside the body and the short length of the access sheath.

Supraclavicular access to IABP placement uses ultrasound guidance to access the subclavian artery and then fluoroscopic assistance is used to help position the tip of IABP in the desired position with full length of the sheath retained. The tip of the sheath is placed in the descending thoracic aorta and could potentially prevent the migration of the device. The left subclavian arterial access is preferred with this approach, as the course of the device is straight from the left side and also reduces the chances of cerebrovascular accident (CVA) [10].

## Mobile extracorporeal membrane oxygenation (ECMO) support

**Fig. 3:**
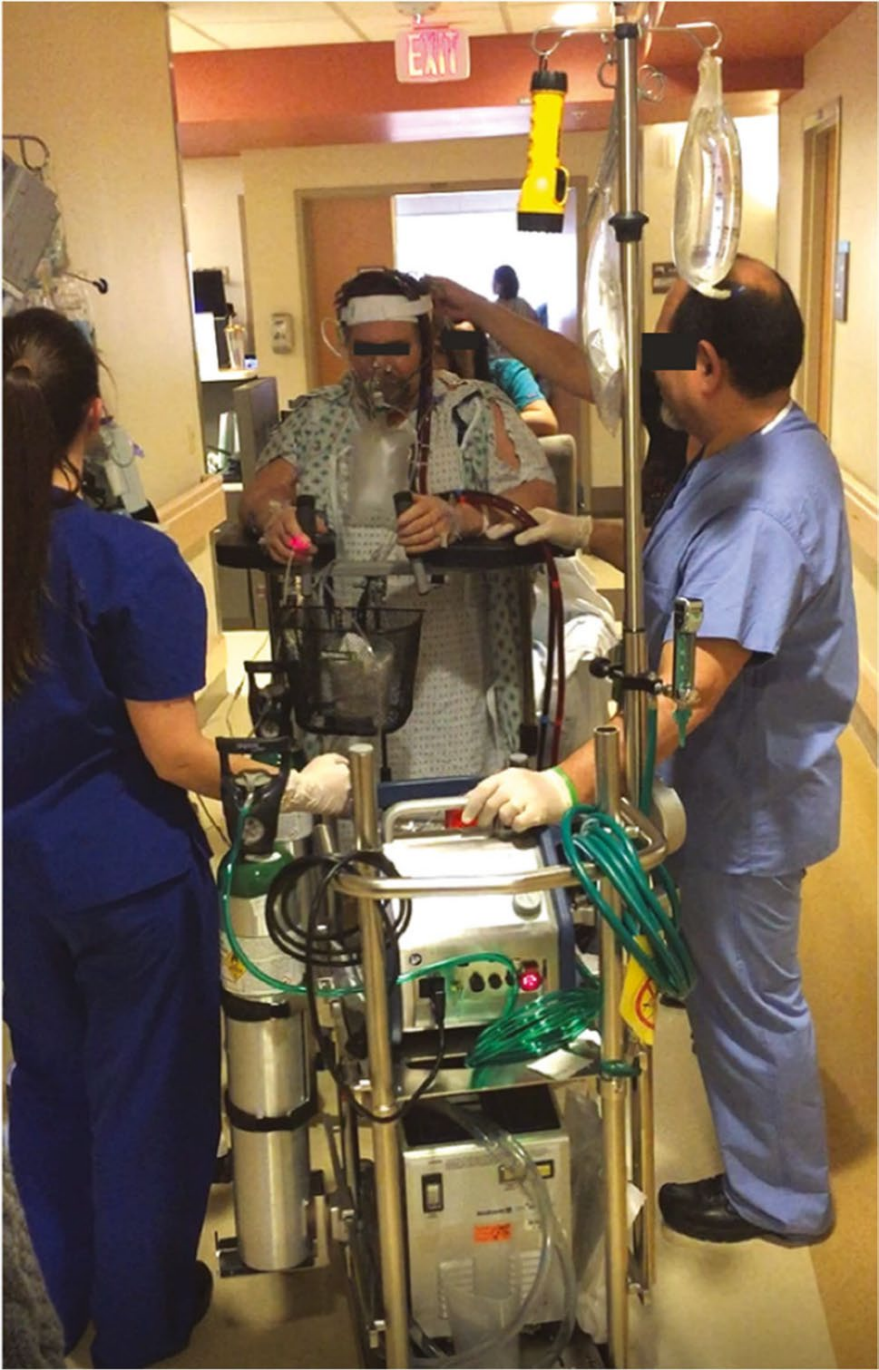
Ambulatory ECMO

Ambulatory veno-venous ECMO using internal jugular venous cannulation has been used more frequently in patients awaiting lung transplantation. Veno-arterial (VA)-ECMO support using alternate access such as the subclavian artery and the subclavian vein or jugular vein is being used in select centers [11]. As cannula design evolves in concert with miniaturization of pumps, ambulatory support is likely to grow in popularity. The large console size of the ECMO circuit requires that the patients need support of the staff to facilitate their ambulation. However, the concept of ambulatory ECMO support has been used in select centers to bridge patients to lung transplantation [12]. The advantage is that while the console maybe bulky, the use of dual-lumen cannulae placed through the internal jugular vein makes it easier for the patient to walk around. Figure 3 is of a patient supported with ECMO, ambulating (courtesy — Dr Suresh Keshavamurthy and IJCTS).

The technique of graft sutured to the axillary artery, tunneled subcutaneously allowing chest closure post cardiotomy, has been utilized in ECMO support. This strategy not only provides the advantage of mechanical stability of the chest wall to allow early extubation but also provides all the advantages of ambulatory MCS (Table 1).

**Table 1:** A comparison of available ambulatory ST-MCS

Device	Indication	Advantages	Disadvantages	Clinical utility
Impella	MCS or support in presence of coronary ischemia	Increased cost with consequent lack of widespread use	Hemolysis Device migration Not well tolerated long-term (> 48–72 h)	LV support; Impella RP for right-sided support Provides effective circulatory support in cardiogenic shock
iVAS	MCS	The pump is small sized and carried as a pack	Risk of late failure and air embolism	LV support. Mild RV support
ECMO	Bi-ventricular MCS	Low cost. Familiarity amongst perfusionists. Relatively easy to manage	Large cannulae Large console Thromboembolic complications Some need for anticoagulation Occasional need for decompression of LV	Used as left-sided and bi-ventricular support, with additional tweaks required for ambulation

## Impella 5.0 and 5.5

Impella is a micro-axial LVAD over a pigtail catheter which is placed into the LV across the aortic valve. The pump provides circulatory support by ejecting the blood from LV into the aorta, thus unloading the ventricle and allowing reduction of LV wall tension. This device provides circulatory support in patients with cardiogenic shock and allows support prior to definitive therapy and as bridge to recovery. Using trans-subclavian approach allows patient improved recovery as they can actively participate in physiotherapy and ambulation. While the 5.0 and 5.5 versions purport to provide 5 and 5.5 L per minute of flow, this is based on algorithms and do not accurately represent actual flows achieved. Furthermore, at high speeds and sometimes with minor changes in device position, there may be higher rates of hemolysis.

### Trans-subclavian approach technique

This was used first by Raman et al. in the University of Chicago in 2010 (unpublished communication) (figure of graft sutured to the subclavian artery) and then adapted to more widespread use [13] as experience grew with the deployment of subclavian access for IABP and transcatheter aortic valve replacement (TAVR) [14].

**Fig. 4:**
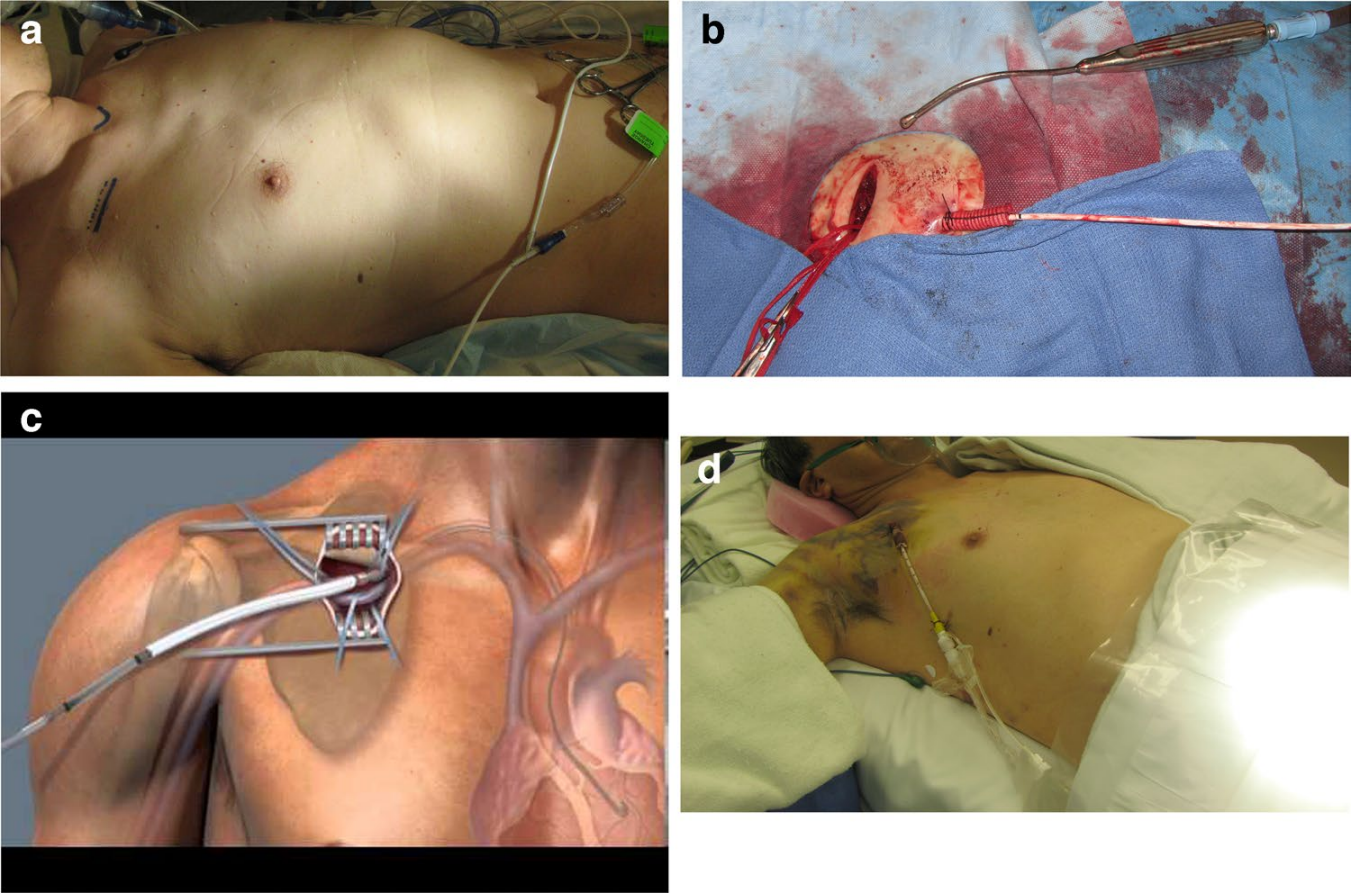
Impella through subclavian artery graft. **a** Skin incision marks in the right anterior chest wall. **b** Graft sutured to the subclavian artery. **c** Schematic of Impella 5.0 in place. **d** Impella in place through graft prior to removal

Usually, a right infraclavicular approach is utilized and a 10-mm Dacron graft is sewn as a chimney to the axillary/subclavian artery [13]. The graft is exteriorized through the incision or via a counter incision. A 23-Fr peeling sheath is inserted into the graft. The sheath is secured by two plastic graft locks. The sheath is de-aired and flushed. A pigtail catheter is advanced into the axillary graft and a 0.035″ J wire is used to advance the device under fluoroscopic guidance in a retrograde fashion. The wire is guided through the graft and is navigated to pass the subclavian/innominate junction into the ascending aorta. The wire and the catheter are placed across the aortic valve into the LV apex. The device is threaded over the guidewire through the graft into the aorta and across the aortic valve. Transesophageal echo (TEE) guidance is used to allow the inlet portion of the device at about 5 cm below the aortic valve. The introductory sheath is removed and the repositioning sheath is advanced at the entry site. Figure 4 illustrates the steps of the Impella 5.0 inserted through a Dacron graft sutured to the subclavian artery. The planning incisions are shown in Fig. 4(a). The 10-mm Dacron graft sutured to the right subclavian artery is depicted in Fig. 4(b). The schematic diagram in Fig. 4(c) depicts the Impella 5.0 in its place with the tip in the left ventricular cavity. Figure 4(d) shows the Impella in place prior to removal after a successful wean off support.

## Other devices

### Intravascular ventricular assist devices (iVAS)

The intravascular ventricular assist system (iVAS) (NuPulseCV, Inc., Raleigh, NC) is a minimally invasive, ambulatory, counterpulsation device which can provide long-term support for patients with advanced HF and can potentially provide support while avoiding the need for a standard VAD. The external driver is a pneumatically driven electromechanical pump. The arterial interface and skin interface devices connect the external and internal drivelines. The authors reported their experience in 13 patients with all patients undergoing cardiac transplant at a mean duration of 32 days [15]. An updated report on this device has suggested 66% of their 45 patients had undergone successful cardiac transplantation at 6 months. There was one mortality and one CVA during this study period [16]. Interestingly, the NuPulse iVAS is a successor to the Kantrowitz CardioVAD [17], which was plagued by air embolism and stroke after 4 to 6 months of implant. If anything, the iVAS experience is very similar, suggesting that the use of compressed air beyond a few weeks is potentially dangerous in terms of air-embolism risk. The device does not preclude the use of a continuous-flow ventricular assist device (cfVAD) if required subsequently to support the circulation. The device conceptually combines the advantages of balloon counter pulsation and cfVAD. As a counterpulsation device, the iVAS augments, but does not replace, a patient’s cardiac output. Thus, a patient must have a certain amount of systolic reserve to benefit from the device. Additionally, there are several contraindications for using iVAS, including an aortic diameter < 20 mm, subclavian diameter < 7 mm, abnormalities of the aorta such as heavy calcification or aneurysms, or uncontrolled arrhythmias that prevent proper electrocardiogram tracing. The device allows the patients to be discharged home, theoretically, with a rather bulky console. The majority of the NuPulse experience is from one center, viz., the University of Chicago, and the patients were confined to hospital for the duration of the support. This might be another instance of a potentially reasonable intervention that fails to gain widespread acceptance due to the limitations of balloon counterpulsation and use of compressed air as the inflating gas. If as the adage goes “Imitation is the best form of flattery”, then the iVAS has failed.

## Conclusions

Management of advanced HF can be challenging. Patients often need hospital-based treatment for titration and monitoring of medical therapy. ST-MCS plays an important role in a subset of these patients who need circulatory support to reverse the cardiac dysfunction and define management pathway in patients who do not improve. Several devices have been available to facilitate patient management. The concept of ambulatory MCS has become popular in the last decade or so. These techniques provide better patient management in the hospital environment allowing more independence to patients who are likely to become malnourished and deconditioned with HF.

Choice of the device depends on the operator and/or institutional experience. None of the systems available is without their own problems. This is an ongoing area of development and future work needs to focus not only on refining the technology but also define the management pathways to tailor a system to the requirement. There is a need to develop reliable devices which may allow the patients to undergo their recuperation in the community.
